# Quality of life in dysphagia and anxiety and depression symptoms pre and post-thyroidectomy

**DOI:** 10.1590/2317-1782/20232022099en

**Published:** 2023-08-07

**Authors:** Gabriel Trevizani Depolli, Gustavo Batista de Oliveira, Thais Jejesky de Oliveira, Marco Homero de Sá Santos, Ricardo Mai Rocha, Michelle Ferreira Guimarães, Elma Heitmann Mares Azevedo

**Affiliations:** 1 Departamento de Fonoaudiologia, Universidade Federal do Espírito Santo - UFES - Vitória (ES), Brasil.; 2 Hospital Universitário Cassiano Antônio Moraes - EBSERH - Vitória (ES), Brasil.; 3 Departamento de Cirurgia de Cabeça e Pescoço, Universidade Federal do Espírito Santo - UFES - Vitória (ES), Brasil.

**Keywords:** Thyroidectomy, Deglutition Disorders, Quality of Life, Anxiety, Depression

## Abstract

**Purpose:**

To correlate the dysphagia quality of life and symptoms of anxiety and depression before and after thyroidectomy.

**Methods:**

Observational, longitudinal, prospective, and experimental study. Twenty patients participated, with a mean age of 54 years, prevalence of females (n=17; 85%) and partial thyroidectomy (n=14; 70%). All subjects underwent laryngeal visual examination and answered the MD Anderson Dysphagia Questionnaire (MDADI) and the Hospital Anxiety and Depression Scale (HADS) in three different moments: preoperatively, immediately postoperatively (maximum one week) and three months after surgery.

**Results:**

There was a significant difference in dysphagia quality of life for the physical and total domains in the three different moments. Regarding anxiety and depression, a statistically significant difference was observed between the scores in all domains, with a greater difference observed between the preoperative period and after 1 week. Higher values were observed in the preoperative period for mild anxiety traits, being more frequent in relation to depression, with a reduction after 1 week and an increase after three months of surgery. There was no significant correlation between the MDADI and HADS protocols.

**Conclusion:**

Patients undergoing thyroidectomy self-report better quality of life in dysphagia and reduced anxiety/depression scores after three months of surgery. There was no correlation between anxiety, depression and quality of life in dysphagia at the moments evaluated.

## INTRODUCTION

Patients diagnosed with benign/malignant thyroid diseases may also have hormonal changes, enlargement of the gland or even thyroid nodules^(1)^. As a result of these conditions, these patients may have dysphagia, either before or after partial or total thyroidectomy^(2-4)^.

There are reports in the literature that patients undergoing thyroidectomy have symptoms suggestive of changes in swallowing, both pre- and postoperatively, characterized by a feeling of dryness, irritated throat, choking and a lump in the throat, which may have an impact in the swallowing function^(5)^ and, consequently, on the patient's quality of life. In addition, complaints related to swallowing and voice before thyroidectomy may be present in up to one- third of patients^(6)^, while the occurrence of symptoms related to swallowing in individuals after thyroidectomy may range from 20% to 58%^(7)^ .

It should be noted that swallowing-related symptoms can cause psychological stress with negative effects on quality of life, considered as a predictor of suffering^(8)^. In this context, anxiety and depression are highly prevalent in patients with thyroid disorders^(9,10)^. Gorkhali et al.^(10)^ reported the presence of anxiety in more than 50% of patients and depression in more than 40%.

A study conducted with patients with thyroid dysfunction showed that even after patients became euthyroid with treatment, they still had a predisposition to anxiety and depression, and more than 50% of patients had mild depression signs after one year of follow-up^(11)^. These symptoms may result from a fear of disease recurrence and progression and influence the individual's quality of life^(12-15)^.

As well as the sensory aspects related to swallowing, emotional issues associated with the diagnosis of thyroid nodules should also be considered in the patient's analysis^(9,16)^. In this sense, the clinical management of thyroid alterations should include the identification and treatment of both swallowing-related symptoms and anxiety and depression, with adequate counseling for these patients^(10)^.

However, there is little strong evidence on the relationship between dysphagia and anxiety/depression and with different populations. A randomized clinical trial with post-stroke dysphagic patients showed different degrees of anxiety/depression^(17)^. Lower anxiety and depression scores, better swallowing function and lower incidence of pneumonia were found in nutritional rehabilitation in post-cerebral infarction patients with alternative intermittent feeding concomitantly with swallowing rehabilitation^(18)^. However, there are still no studies addressing the quality of life in dysphagia and emotional symptoms in patients undergoing thyroidectomy longitudinally.

Therefore, the investigation of quality of life in dysphagia and symptoms of anxiety and/or depression can help in early clinical management, improving the patient's prognosis.

Thus, this study aimed to correlate quality of life in dysphagia and symptoms of anxiety/depression before and after thyroidectomy.

## METHODS

This was an observational, longitudinal, prospective and quantitative study approved by the Research Ethics Committee of the Institution under the Decision No. 2.644.055. All study participants signed the Informed Consent Form.

The study included 20 patients with thyroid disease, with a mean age of 54 years (standard deviation= ±16.9), and a higher prevalence of females (n=17; 85%) and partial thyroidectomy (n=14; 70%) . The sample was selected for convenience and the participants were treated at the Head and Neck Surgery Department of a University Hospital. In turn, the study excluded patients with reduced alertness or cognitive status, with other oncological diagnoses, who had undergone non-surgical treatments, with a neurological diagnosis, as well as patients with other oncological diagnoses, who have undergone non-surgical treatments, with a neurological diagnosis, patients who had laryngeal alterations and endolaryngeal signs of laryngopharyngeal reflux, visualized through videolaryngoscopy, performed in the three moments, such as the presence of hyperemia and edema of the posterior third of the glottic and interarytenoid region (diagnosis dependent on medical evaluation without specific criteria), and data on thyroid hormone alterations obtained by measuring TSH (thyroid-stimulating hormone) and free T4 in the blood (reference value for TSH: 0.27 to 4.2, and for Free T4: 0.93 to 1.710), requested on a regular basis in the outpatient follow-up of the patients and collected from the medical records in all three moments.

The patients completed the M.D. Anderson Dysphagia Inventory (MDADI)^(19,20)^ and the Hospital Anxiety and Depression Scale (HADS)^(21,22)^ at three different times: (a) Preoperative period; (b) Recent postoperative period (maximum of one week); and (c) after three months. The MDADI allows patients to self-evaluate their perception of their swallowing function after treatment for head and neck cancer and its impact on their quality of life. The questionnaire includes 20 questions, one of which is a global question and the others subdivided into domains: emotional (6 questions), functional (5 questions) and physical (8 questions). Scores between 0 and 100 are assigned to all domains and the lower the score, the worse the effect of dysphagia on quality of life. The total score is obtained by the total average of the domains, in which values from 0 to 20 are considered as profound limitation, 21 to 40 for severe limitation, 41 to 60 for moderate limitation, 61 to 80 for medium limitation and 81 to 100 for minimal limitation^(20)^.

In turn, HADS allows the assessment of mood disorders in patients with physical illnesses. This tool includes 14 multiple-choice questions divided into two subscales, one for anxiety and the other for depression, with seven items each. The overall score on each subscale ranges from 0 to 21. Anxiety and depression scores are categorized into normal (0-7), mild (8-10), moderate (11-14), and severe (15-21). A cutoff of 8 or more points represents possible cases of anxiety/depression, while 11 or more points represent probable cases of anxiety/depression^(21,22)^.

All data collection was carried out through interviews by the researcher, who has experience in the area of dysphagia and in the application of self-assessment protocols.

Descriptive statistics of the instruments used were performed for data analysis. The Friedman test was used to compare the pre- and post-surgery periods and the Conover Post-hoc Test to precisely identify at which time points in the study this difference occurred. Spearman's rank correlation coefficient was used to measure the correlation between instrument scores. A significance level of 5% (p-value ≤ 0.05) was adopted for all analyzes. Aiming at analyzing the magnitudes of correlation, values below 0.50 were considered weak, while values between 0.50 and 0.7 were considered moderate, between 0.70 and 0.90 were considered strong and values above 0.90 were considered very strong^(23)^. All statistical analyzes were performed using the R v3.6.1.

## RESULTS

There was a prevalence of average limitation for the emotional and functional domains in the three time periods. The physical domain showed moderate limitation in the preoperative and recent postoperative periods. In addition, there was a significant difference in the “physical” and “total” domains of the MDADI between the preoperative period and three months after (“a” and “c”), as well as for the recent postoperative period and three months after (“b” and “c”). (Table 1).

**Table 1:** Comparison of the median of the scores of MDADI domains, at different times, in patients undergoing thyroidectomy

***MDADI***	**Periods**			**p-value**
	Pre	After 1W	After 3M	
Overall	4.0	4.0	4.0	0.359
Emotional	71.7	71.7	78.0	0.081
Functional	70.0	66.0	73.8	0.711
Physical	58.8^c^	55.0^c^	74.9^a^^,^^b^	<0.001**
Total	67.7^c^	62.8^c^	74.2^a,b^	<0.047**

a Statistically significant difference when compared to the preoperative period;

b Statistically significant difference when compared to the recent postoperative period;

c Statistically significant difference when compared to the three months postoperative period;

** Friedman test; 95% Confidence Interval; p ≤ 0.05; p ≤ 0.05; Conover Post-hoc Test (p-value ≤ 0.05) at different times: Pre (a), Recent postoperative period (b) and After 3M (c)

**Caption:** MDADI = MD Anderson Dysphagia Questionnaire; Pre = Preoperative Period; After 1W = Recent Postoperative Period of a Maximum of One Week; After 3M = After Three Months

The greatest difference in the HADS scale, both for anxiety and depression, was found between the preoperative period (a) and the recent postoperative period (b), and the values found in the preoperative period were characterized as “mild traits” for anxiety and “normal traits” for depression. In addition, there was a statistically significant difference between all pairs of time periods in the “anxiety” domain and in the total score of the HADS scale. There was a statistically significant difference in the “Depression” domain between the preoperative and early postoperative period (1W) (“a” and “b”) and the preoperative period and 3 months after surgery (3M) (“a” and “c”) (Table 2).

**Table 2:** Comparison of the scores of HADS domains, at different times, in patients undergoing thyroidectomy

***HADS***	**Periods**			**p-value**
	Pre	After 1W	After 3M	
Anxiety	8.1^b^^,^^c^	5.0^a^^,c^	6.7^a,b^	0. 012**
Depression	5.3^b,c^	3.7^a^	4.1^a^	0. 010**
Total	13.5^b,c^	8.7^a,c^	10.8^a,b^	0. 014**

a Statistically significant difference when compared to the preoperative period;

b Statistically significant difference when compared to the recent postoperative period;

c Statistically significant difference when compared to the three months postoperative period;

** Friedman test (p ≤ 0.05); 95% Confidence Interval; Conover Post-hoc Test (p-value ≤ 0.05) at different times: Pre (a), After 1W (b) and After 3M (c)

**Caption:** HADS = Hospital Anxiety and Depression Scale; Pre = Preoperative Period; After 1W = Recent Postoperative Period of a Maximum of One Week; After 3M = After Three Months

No statistically significant correlation was found between the domains of the HADS scale and the MDADI questionnaire at the evaluated periods (Table 3).

**Table 3:** Correlation between MDADI domain scores and HADS items, at different times, in patients undergoing thyroidectomy

***MDADI***	***HADS-A***			***HADS-D***			***HADS-T***		
	Pre	After 1W	After 3M	Pre	After 1W	After 3M	Pre	After 1W	After 3M
Overall	-0.145	-0.062	0.194	0.004	-0.281	0.206	-0.102	-0.152	0.222
Emotional	0.019	-0.034	0.139	0.087	-0.296	0.143	0.058	-0.150	-0.174
Functional	0.069	0.005	0.359	0.015	-0.117	0.310	0.010	-0.054	0.347
Physical	-0.179	-0.320	0.217	-0.141	-0.388	0.141	-0.226	-0.389	0.231
Total	-0.140	-0.132	0.162	-0.103	-0.297	0.118	-0.176	-0.211	0.175

**Caption:** Spearman's rank correlation coefficient (p ≤ 0.05); 95% Confidence Interval; MDADI = M.D. Anderson Dysphagia Inventory; HADS-A = Anxiety Domain of the Hospital Anxiety and Depression Scale; HADS-D = Depression Domain of the Hospital Anxiety and Depression Scale; HADS-T = Total Domain of the Hospital Anxiety and Depression Scale; Pre = Preoperative Period; After 1W = Recent Postoperative Period of a Maximum Of One Week; After 3M = After Three Months

## DISCUSSION

Patients submitted to thyroidectomy may have vocal and swallowing alterations even with the preservation of the laryngeal nerves^(3,4,24)^, which impact the individual's quality of life and emotional state^(25)^. The understanding and identification of these parameters helps in clinical decision-making and in better communication in the professional-patient relationship.

The MDADI results showed a difference in the three periods analyzed for the “physical” and “total” domains. In addition, scores improved in all domains after three months of surgery, except for overall, in which the median score was maintained (Table 1). Another study concluded that there was an improvement in the quality of life related to swallowing of the participants after six months of treatment and that symptoms related to dysphagia in the recent postoperative period reduced during follow-up^(4)^.

The study by Gumus et al.^(4)^ found a difference only for the “total” domain score of the MDADI, which made it impossible to compare all domains with the data from this study. Therefore, it is suggested to carry out studies investigating the different domains of MDADI in patients with thyroid alterations, facilitating the understanding of the different factors of quality of life in dysphagia.

The statistically significant difference found in this study between the “physical” and “total” MDADI domains is in line with a Brazilian study carried out with patients with thyroid carcinoma, which found an improvement in the scores of all MDADI domains after three months of treatment with radioiodine therapy^(26)^. Although the treatment method was different from the treatment of the participants in this study, the difference between the scores of the domains in both studies strengthens the hypothesis that both thyroidectomy and radioiodine therapy can improve the quality of life related to swallowing in patients with thyroid disorders. Furthermore, it should be noted the difficulty of finding studies investigating the quality of life related to post-thyroidectomy swallowing.

The “physical” domain proved to be the most affected in the preoperative and post-operative moments. Regarding the difference between the periods for this domain, it is known that even without manipulation and/or injury to the recurrent laryngeal nerve, voice and/or swallowing changes can be self-reported by patients undergoing thyroidectomy^(4)^. Therefore, it is believed that the “physical” domain has a greater impact when compared to the other domains.

As for the HADS scale, the patients had scores characterized as mild anxiety traits and greater than depression traits. These scores were higher in the preoperative period, decreasing in the recent postoperative period and increasing three months after surgery (Table 2).

Although the traits have been considered mild for anxiety and depression, these symptoms are frequent in patients with thyroid alterations^(9,10)^ and should be evaluated and included in the scope of the management of patients with such alterations^(10)^, and should be analyzed longitudinally. Furthermore, the literature indicates that dysphagia is a predictor of depression^(27)^.

Regarding anxiety, there was a difference between all analyzed moments, while for depression, the only period that did not present a significant difference was between the recent postoperative period and the three months after (Table 2). These findings show the need for multidisciplinary follow-up, including psychological assistance, from the beginning of the treatment, aiming to minimize the emotional impact that the difficulty in swallowing can cause through strategies such as cognitive-behavioral psychotherapy, which has been shown to be effective in the follow-up of patients with thyroid disease^(28)^.

As shown in Table 3, there was no correlation between MDADI and HADS scores. However, correlations of both questionnaires have already been found in other studies, with different populations, such as in patients submitted to total laryngectomy^(29)^ and in patients who survived head and neck cancer in general^(30)^. The non-correlation between the scores of the applied questionnaires may have occurred due to the sample size or the uniqueness of the patients' responses and/or several other sampling factors. Therefore, studies are suggested to investigate the correlation between both questionnaires in the population with thyroid disorders in the long term.

Some limitations were identified in this study, such as the reduced sample size due to the suspension of outpatient care, due to the COVID-19 pandemic, in addition to the difficulty of contact by telephone, lack of important information about the reasons for which the patients underwent to thyroidectomy and further detailing of the sample with data referring to thyroid disease, such as the type of lesion, lack of data on psychiatric diagnosis of any mental disorder, use of medication such as anxiolytics and lack of criteria for considering the presence of edema, which could reflect on clinical implications and greater consistency of the discussion.

## CONCLUSION

Patients undergoing thyroidectomy self-report better quality of life in dysphagia and reduced anxiety/depression scores after three months of surgery. There was no correlation between anxiety, depression and quality of life in dysphagia at the evaluated periods.
