# Supplementary material for: Quality of life in dysphagia and anxiety and depression symptoms pre and post-thyroidectomy
Source: Codas. 2023 Aug 7;35(5):e20220099. doi: 10.1590/2317-1782/20232022099en (PMC10449093; doi:10.1590/2317-1782/20232022099en)
Supplement: Translation

# Translation: Qualidade de vida em disfagia e sintomas de ansiedade e depressão pré e pós-tireoidectomia

**DOI:** 10.1590/2317-1782/20232022099pt

**Authors:** Gabriel Trevizani Depolli, Gustavo Batista de Oliveira, Thais Jejesky de Oliveira, Marco Homero de Sá Santos, Ricardo Mai Rocha, Michelle Ferreira Guimarães, Elma Heitmann Mares Azevedo

**Affiliations:** 1 Departamento de Fonoaudiologia, Universidade Federal do Espírito Santo - UFES - Vitória (ES), Brasil.; 2 Hospital Universitário Cassiano Antônio Moraes - EBSERH - Vitória (ES), Brasil.; 3 Departamento de Cirurgia de Cabeça e Pescoço, Universidade Federal do Espírito Santo - UFES - Vitória (ES), Brasil.

**Keywords:** Tireoidectomia, Transtornos de Deglutição, Qualidade de Vida, Ansiedade, Depressão

## Abstract

**Objetivo:**

Correlacionar a qualidade de vida em disfagia e sintomas de ansiedade e depressão pré e pós-tireoidectomia.

**Método:**

Estudo observacional, longitudinal, prospectivo e quantitativo. Participaram 20 pacientes, com média de idade de 54 anos, maior prevalência do sexo feminino (n=17; 85%) e de tireoidectomia parcial (n=14; 70%). Todos realizaram exame visual laríngeo e responderam ao Questionário de Disfagia M. D. Anderson (*MDADI*) e à Escala Hospitalar de Ansiedade e Depressão (*HADS*), no pré-operatório, pós-operatório recente (máximo uma semana) e três meses pós-operatório.

**Resultados:**

Houve diferença significante na qualidade de vida em disfagia, para os domínios físico e total, nos três momentos. Quanto à ansiedade e depressão foi observada diferença estatisticamente significante entre as pontuações em todos os domínios, com maior diferença observada entre o pré-operatório e pós-operatório recente. Foram observados valores mais altos no pré-operatório para traços leves de ansiedade, sendo mais frequentes em relação à depressão, com redução após 1 semana e aumento após três meses de cirurgia. Não houve correlação significante entre os protocolos *MDADI* e *HADS*.

**Conclusão:**

Os pacientes submetidos à tireoidectomia autorreferem melhor qualidade de vida em disfagia e redução dos escores de ansiedade/depressão após três meses de cirurgia. Não houve correlação entre ansiedade, depressão e qualidade de vida em disfagia nos momentos avaliados.

## INTRODUÇÃO

Pacientes diagnosticados com doenças benignas/malignas tireoidianas podem apresentar alterações hormonais, aumento da glândula ou ainda nódulos na tireoide^(1)^. Consequentemente, esses pacientes podem desenvolver disfagia, antes ou após a tireoidectomia, seja ela parcial ou total^(2-4)^.

Segundo a literatura, pacientes submetidos à tireoidectomia apresentam sintomas sugestivos de alterações na deglutição, tanto no pré quanto no pós-operatório, caracterizados por sensação de secura, garganta irritada, engasgos e bola na garganta, que podem impactar a função da deglutição^(5)^ e, consequentemente, a qualidade de vida do paciente. Além disso, queixas relacionadas à deglutição e à voz antes da tireoidectomia podem ocorrer em um terço dos pacientes^(6)^ e a ocorrência de sintomas relacionados à deglutição em indivíduos pós-tireoidectomia pode variar entre 20% e 58%^(7)^.

Sintomas relacionados à deglutição podem gerar estresse psicológico com efeitos negativos na qualidade de vida, considerado como preditor de sofrimento^(8)^. A ansiedade e a depressão são altamente prevalentes em pacientes com alterações tireoidianas^(9,10)^. Para Gorkhali et al.^(10)^, a ansiedade é percebida em mais de 50% dos pacientes e a depressão em mais de 40%.

Um estudo realizado com pacientes com disfunção tireoidiana observou que, mesmo após os pacientes se tornarem eutireoidianos com o tratamento, eles ainda apresentaram predisposição à ansiedade e depressão e mais de 50% apresentaram traços de depressão considerados leves após um ano de *follow-up* ^(11)^. Esses sintomas podem ser explicados pelo medo de recidiva e progressão da doença e influenciam na qualidade de vida do indivíduo^(12-15)^.

Além de aspectos sensoriais relacionados à deglutição deve-se considerar questões emocionais associadas ao diagnóstico dos nódulos tireoidianos^(9,16)^ e, à vista disso, no manejo clínico de alterações tireoidianas, deve-se incluir a identificação e o tratamento tanto de sintomas relacionados à deglutição como de ansiedade e depressão, com aconselhamento adequado a esses pacientes^(10)^.

Existem poucas evidências fortes sobre a relação entre a disfagia e a ansiedade/depressão e com populações diferentes. Um estudo clínico randomizado com pacientes disfágicos pós-derrame mostra diferentes graus de ansiedade/depressão^(17)^. A reabilitação nutricional com via alternativa de alimentação intermitente concomitante à reabilitação de deglutição, em pacientes pós-infarto cerebral, demonstra menores escores de ansiedade e depressão, melhor função de deglutição e menor incidência de pneumonia^(18)^. No entanto, até o momento, não há algum estudo que aborde qualidade de vida em disfagia e sintomas emocionais em pacientes submetidos à tireoidectomia de forma longitudinal.

Sendo assim, investigar qualidade de vida em disfagia e sintomas de ansiedade e/ou depressão pode auxiliar na precocidade do manejo clínico, melhorando o prognóstico do paciente.

Desta forma, o presente estudo pretendeu correlacionar a qualidade de vida em disfagia e sintomas de ansiedade/depressão pré e pós-tireoidectomia.

## MÉTODO

Tratou-se de um estudo observacional, longitudinal, prospectivo e quantitativo, com aprovação do Comitê de Ética e Pesquisa (CEP) da Instituição de origem, sob o nº 2.644.055. Todos os indivíduos que participaram do estudo assinaram o Termo de Consentimento Livre e Esclarecido.

Foram incluídos 20 pacientes com doença tireoidiana, com média de idade de 54 anos (desvio padrão= ±16,9), maior prevalência de sexo feminino (n=17; 85%) e tireoidectomia parcial (n=14; 70%). A amostra foi por conveniência e os participantes foram atendidos no Setor de Cirurgia de Cabeça e Pescoço de um Hospital Universitário. Foram excluídos pacientes com redução do estado de alerta ou do estado cognitivo, com outros diagnósticos oncológicos, que tenham sido submetidos a tratamentos não cirúrgicos, com diagnóstico neurológico pacientes que possuíam alterações laríngeas e sinais endolaríngeos de refluxo laringofaríngeo, visualizados por meio de videolaringoscopia, realizada nos três momentos, como presença de hiperemia e edema de terço posterior da região glótica e interaritenoidea (diagnóstico dependente da avaliação médica sem critério específico), e dados de alterações hormonais tireoidianas obtidas pela dosagem de TSH (hormônio tireoestimulante) e T4 livre no sangue (valor de referência para TSH: 0,27 a 4,2 e para T4livre: 0,93 a 1,710), solicitadas de maneira regular no seguimento ambulatorial dos pacientes e colhidas dos prontuários em todos os três momentos.

Os pacientes responderam as versões brasileiras do Questionário de Disfagia M. D. Anderson (*MDADI*)^(19,20)^ e da Escala Hospitalar de Ansiedade e Depressão (*Hospital Anxiety and Depression Scale*-*HADS*)^(21,22)^ em três diferentes momentos: (a) pré-operatório; (b) pós-operatório recente (máximo 1 semana após a cirurgia); (c) três meses pós-operatório. O *MDADI* permite a autoavaliação dos pacientes quanto à percepção de sua função de deglutição após o tratamento do câncer de cabeça e pescoço e o quanto isso afeta a sua qualidade de vida. O questionário contém 20 questões, sendo uma global e outras subdivididas nos domínios: emocional (6 questões), funcional (5 questões) e físico (8 questões). Todos os domínios são pontuados entre 0 e 100 e quanto menor a pontuação, pior o efeito da disfagia na qualidade de vida. Seu escore total é formado pela média total dos domínios, sendo considerados os valores de 0 a 20 para limitação profunda, 21 a 40 para limitação grave, 41 a 60 para limitação moderada, 61 a 80 para limitação média e 81 a 100 para limitação mínima^(20)^.

A *HADS* permite a avaliação de transtornos do humor em pacientes com doenças físicas. Contém 14 questões do tipo múltipla escolha divididas em duas subescalas, uma para ansiedade e outra para depressão, com sete itens em cada. A pontuação global em cada subescala varia entre zero e 21. Os escores de ansiedade e depressão são categorizados em normal (0-7), leve (8-10), moderado (11-14) e grave (15-21). O ponto de corte de 8 ou mais identifica casos possíveis de ansiedade e/ou depressão e pontuações iguais ou superiores a 11 casos prováveis de ansiedade/depressão^(21,22)^.

Toda a coleta de dados foi realizada como forma de entrevista pela pesquisadora responsável, que possui experiência na área de disfagia e na aplicação de protocolos de autoavaliação.

Para análise dos dados, foi realizada estatística descritiva dos instrumentos utilizados. O Teste de Friedman foi utilizado para comparação dos momentos pré e pós-cirurgia e o Teste Post-hoc de Conover para identificar com precisão em quais momentos do estudo ocorreu essa diferença. O Teste de Correlação de Spearman foi utilizado para medir a correlação entre as pontuações dos instrumentos. Foi adotado o nível de significância de 5% (p-valor ≤0,05). Para análise das magnitudes de correlação, valores de correlação abaixo de 0,50 foram consideradas fracas, entre 0,50 e 0,7 consideradas moderadas, entre 0,70 e 0,90 consideradas fortes e acima de 0,90 consideradas muito fortes^(23)^. Todas as análises estatísticas foram realizadas no programa R versão 3.6.1.

## RESULTADOS

Nos três momentos houve prevalência de limitação média para os domínios emocional e funcional. O domínio físico apresentou limitação moderada no pré e pós-recente. Além disso, houve diferença significante nos domínios “físico” e “total” do *MDADI* entre o momento pré-operatório e pós três meses (“a” e “c”) e o pós-operatório recente e pós- três meses (“b” e “c”). (Tabela 1).

**Tabela 1:** Comparação das medianas dos escores dos domínios do *MDADI* nos diferentes momentos em pacientes submetidos à tireoidectomia

***MDADI***	**Momentos**			**p-valor**
	Pré	Pós 1S	Pós 3M	
Global	4,0	4,0	4,0	0,359
Emocional	71,7	71,7	78,0	0,081
Funcional	70,0	66,0	73,8	0,711
Físico	58,8^c^	55,0^c^	74,9^a^^,^^b^	<0,001**
Total	67,7^c^	62,8^c^	74,2^a,b^	<0,047**

a Diferença estatisticamente significativa quando comparada ao momento pré;

b Diferença estatisticamente significativa quando comparada ao momento pós-operatório recente;

c Diferença estatisticamente significativa quando comparada ao momento pós 3M;

** Teste de Friedman; Intervalo de confiança de 95%; p ≤ 0,05; p ≤ 0,05; Teste Post-hoc de Conover (p-valor ≤ 0,05) entre os momentos Pré (a), Pós-operatório recente (b) e Pós 3M (c)

**Legenda:** MDADI = Questionário de Disfagia M. D. Anderson; Pré = Pré-operatório; Pós 1S = Pós-operatório Recente de no Máximo Uma Semana; Pós 3M = Pós Três Meses

Na escala *HADS*, tanto para ansiedade quanto para a depressão a maior diferença foi observada entre o pré-operatório (a) e o pós-operatório recente (b), sendo os valores encontrados no pré-operatório caracterizados como “traços leves” de ansiedade e “traços normais” para depressão. Além disso, houve diferença estatisticamente significante entre todos os pares de momentos no domínio “ansiedade” e no escore total da escala HADS. No domínio “Depressão” houve diferença estatisticamente significante entre os momentos pré e pós-operatório recente (1S) (“a” e “b”) e pré e pós 3 meses (3M) (“a” e “c”) (Tabela 2).

**Tabela 2:** Comparação dos escores dos domínios da *HADS* nos diferentes momentos em pacientes submetidos à tireoidectomia

***HADS***	**Momentos**			**p-valor**
	Pré	Pós 1S	Pós 3M	
Ansiedade	8,1^b^^,^^c^	5,0^a^^,c^	6,7^a,b^	0,012**
Depressão	5,3^b,c^	3,7^a^	4,1^a^	0,010**
Total	13,5^b,c^	8,7^a,c^	10,8^a,b]^	0,014**

a Diferença estatisticamente significativa quando comparada ao momento pré;

b Diferença estatisticamente significativa quando comparada ao momento pós-operatório recente;

c Diferença estatisticamente significativa quando comparada ao momento pós 3M;

** Teste de Friedman (p ≤ 0,05); Intervalo de confiança de 95%; Teste Post-hoc de Conover (p-valor ≤ 0,05) entre os momentos Pré (a), Pós 1S (b) e Pós 3M (c)

**Legenda:***HADS* = Escala Hospitalar de Ansiedade e Depressão; Pré = Pré-operatório; Pós 1S = Pós-operatório Recente de no Máximo Uma Semana; Pós 3M= Pós Três Meses

Não houve correlação estatisticamente significante entre os domínios da escala *HADS* e o questionário *MDADI* nos momentos avaliados (Tabela 3).

**Tabela 3:** Correlação entre os escores dos domínios do *MDADI* e os itens da *HADS* nos diferentes momentos em pacientes submetidos à tireoidectomia

***MDADI***	***HADS-A***			***HADS-D***			***HADS-T***		
	Pré	Pós 1S	Pós 3M	Pré	Pós 1S	Pós 3M	Pré	Pós 1S	Pós 3M
Global	-0,145	-0,062	0,194	0,004	-0,281	0,206	-0,102	-0,152	0,222
Emocional	0,019	-0,034	0,139	0,087	-0,296	0,143	0,058	-0,150	-0,174
Funcional	0,069	0,005	0,359	0,015	-0,117	0,310	0,010	-0,054	0,347
Físico	-0,179	-0,320	0,217	-0,141	-0,388	0,141	-0,226	-0,389	0,231
Total	-0,140	-0,132	0,162	-0,103	-0,297	0,118	-0,176	-0,211	0,175

**Legenda:** Teste de Correlação de Spearman (p ≤ 0,05); Intervalo de confiança de 95%; *MDADI* = Questionário de Disfagia M. D. Anderson; *HADS-A* = Domínio de Ansiedade da Escala Hospitalar de Ansiedade e Depressão; *HADS-D* = Domínio de Depressão da Escala Hospitalar de Ansiedade e Depressão; *HADS-T* = Domínio Total da Escala Hospitalar de Ansiedade e Depressão; Pré = Pré-operatório; Pós 1S = Pós-operatório Recente de no Máximo Uma Semana; Pós 3M = Pós Três Meses

## DISCUSSÃO

Pacientes submetidos à tireoidectomia podem apresentar alterações vocais e de deglutição mesmo na presença de preservação dos nervos laríngeos^(3,4,24)^, que impactam a qualidade de vida do indivíduo e seu estado emocional^(25)^. A compreensão e identificação de tais parâmetros auxilia na tomada de decisão clínica e na melhor comunicação entre profissional/paciente.

Os resultados do *MDADI* demonstraram que houve diferença nos três momentos analisados para os domínios “físico” e “total”. E, após três meses de cirurgia, os escores melhoraram em todos os domínios, exceto para o global, no qual a mediana do escore se manteve (Tabela 1). Um outro estudo concluiu que sua amostra apresentou melhora da qualidade de vida relacionada à deglutição após seis meses de tratamento e que os sintomas relacionados à disfagia no período pós-operatório recente reduziram durante o acompanhamento^(4)^.

No estudo de Gumus et al.^(4)^ houve diferença apenas para o escore do domínio “total” do *MDADI*, inviabilizando a comparação de todos os domínios com os dados do presente estudo. Por isso, sugere-se estudos que analisem os diferentes domínios do *MDADI* em pacientes com alterações tireoidianas, facilitando a compreensão dos diferentes fatores da qualidade de vida em disfagia.

A diferença estatisticamente significante encontrada neste estudo, entre os domínios “físico” e total” do *MDADI*, corrobora um estudo brasileiro realizado com pacientes com carcinoma de tireoide, no qual que se verificou melhora nos escores de todos os domínios do *MDADI* após três meses de tratamento com radioiodoterapia^(26)^. Embora o método de tratamento tenha sido diferente do tratamento dos participantes desta pesquisa, a diferença entre os escores dos domínios, em ambos os estudos, fortalece a hipótese de que tanto a tireoidectomia quanto a radioiodoterapia podem melhorar a qualidade de vida relacionada à deglutição de pacientes com alterações tireoidianas. Ademais, salienta-se a dificuldade de se encontrar estudos que tenham analisado a qualidade de vida relacionada à deglutição pós-tireoidectomia.

Observou-se que o domínio “físico” foi o mais afetado nos momentos pré e pós-recente. Em relação à diferença entre os momentos para esse domínio, sabe-se que mesmo sem manipulação e/ou lesão do nervo laríngeo recorrente, alterações de voz e/ou de deglutição podem ser autorreferidas pelos pacientes submetidos à tireoidectomia^(4)^. Por isso, acredita-se que o domínio “físico” apresenta maior impacto quando comparado aos demais domínios.

Quanto à escala *HADS*, os pacientes apresentaram escores caracterizados como traços leves de ansiedade e superiores aos traços de depressão. Esses escores foram maiores no pré-operatório, reduzindo no pós-recente e aumentando pós três meses de cirurgia (Tabela 2).

Embora os traços tenham sido considerados leves para ansiedade e depressão, esses sintomas são frequentes em pacientes com alterações tireoidianas^(9,10)^ e devem ser avaliados e fazer parte do escopo no manejo de pacientes com tais alterações^(10)^ e analisados longitudinalmente. Além disso, a literatura ressalta a disfagia como um preditor de depressão^(27)^.

A ansiedade apresentou diferença entre todos os momentos analisados, enquanto a depressão só não apresentou diferença significante entre o pós-recente e o pós três meses (Tabela 2). Esses achados demonstram a necessidade do acompanhamento multiprofissional, incluindo a assistência da psicologia, desde o início do tratamento para minimizar o impacto emocional que a dificuldade para deglutir pode acarretar por meio de estratégias como a psicoterapia cognitivo-comportamental que tem demonstrado ser eficaz no acompanhamento de pacientes com doenças da tireoide^(28)^.

Não houve correlação entre os escores do *MDADI* e da *HADS* (Tabela 3). Entretanto, ambos os questionários já apresentaram correlações em outros estudos, com populações diferentes, como em pacientes submetidos à laringectomia total^(29)^ e em pacientes sobreviventes de câncer de cabeça e pescoço em geral^(30)^. A não correlação entre os escores dos questionários aplicados pode ter ocorrido pelo tamanho da amostra ou a singularidade das respostas dos pacientes e/ou por diversos outros fatores amostrais. Por isso, sugere-se estudos que investiguem a correlação entre ambos os questionários na população com alterações tireoidianas no longo prazo.

Algumas limitações foram identificadas no estudo, como o tamanho reduzido da amostra devido à suspensão do ambulatório pela pandemia por COVID-19, a dificuldade de contato por telefone, ausência de informações importantes sobre os motivos pelos quais os pacientes foram submetidos à tireoidectomia e maior detalhamento da amostra com dados referentes à doença tireoidiana, como o tipo de lesão, ausência de dados sobre diagnóstico psiquiátrico de algum transtorno mental, uso de medicamentos como ansiolíticos e ausência de critérios para consideração da presença de edema, os quais poderiam refletir em implicações clínicas e maior robustez da discussão.

## CONCLUSÃO

Os pacientes submetidos à tireoidectomia autorreferem melhor qualidade de vida em disfagia e redução dos escores de ansiedade/depressão após três meses de cirurgia. Não houve correlação entre ansiedade, depressão e qualidade de vida em disfagia nos momentos avaliados.
